# Human-Origin Influenza A(H3N2) Viruses Revealed in Swine Farms During the Period 2022–2025 in Kazakhstan

**DOI:** 10.3390/ani16111752

**Published:** 2026-06-05

**Authors:** Nailya Klivleyeva, Tatyana Glebova, Nurbol Saktaganov, Assem Baimukhametova, Mereke Kalkozhayeva, Nuray Ongarbayeva, Indira Ibragimova, Primkul Ibragimov, Richard Webby

**Affiliations:** 1 The Research and Production Center for Microbiology and Virology, Almaty 050010, Kazakhstan; 2 Postgraduate Cardiology Course, Kazakhstan-Russian Medical University, Almaty 050000, Kazakhstan; 3 Non-Profit Joint-Stock Company, Kazakh National Agrarian Research University, Almaty 050010, Kazakhstan; 4 Department of Infectious Diseases, St. Jude Children’s Research Hospital, Memphis, TN 38105-3678, USA

**Keywords:** virus, influenza viruses, swine influenza, A(H3N2) virus, zoonotic infection, Kazakhstan, pandemic risk

## Abstract

Seasonal human influenza A viruses (H1N1 and H3N2) are frequently transmitted to swine populations. Continued transmission of these viruses among swine results in genetic and phenotypic changes, creating new lineages of swine influenza viruses that differ from their parent strains. This contributes to increased viral genetic diversity and the emergence of zoonotic risks. The aim of this study was to assess the prevalence of influenza viruses, including presumably human-origin A(H3N2) strains, on swine farms in Kazakhstan from 2022 to 2025. Real-time RT-PCR was used to test nasal swab antigens, and hemagglutinin inhibition test, and ELISA were used to determine serum antibody levels. Influenza A/H1N1 and A/H3N2 viruses, as well as the A(H7) hemagglutinin genes, were detected in swine. Serological analysis confirmed the presence of antibodies to the A/H1N1pdm09 and A/H3N2 viruses. The influenza A(H3N2) isolates were found to be genetically similar to human viruses of the J.2 or 3C.2a1b.2a.2a.3a.1 clade, possibly indicating transmission from humans to animals. The ongoing exchange of influenza viruses between humans and swine poses a threat of future pandemics. These studies highlight the need for continuous monitoring of swine influenza in Kazakhstan to understand virus evolution and protect public health.

## Simple Summary

The introduction of seasonal human H1N1 and H3N2 influenza viruses into swine populations occurs regularly. As these viruses circulate among swine, they evolve, becoming antigenically distinct from their parent strains, contributing to genetic diversity and the emergence of new swine influenza virus lineages and associated zoonotic risks. The objective of this study was to assess the prevalence of influenza viruses, including A(H3N2), presumed to be of human origin, detected on swine farms in Kazakhstan in 2022–2025. Nasal swab and serum samples from swine were used. The presence of influenza virus antigens in nasal swabs was analyzed using real-time RT-PCR. The levels of specific antibodies in serum were determined by hemagglutination inhibition and enzyme-linked immunosorbent assay. Influenza A/H1N1 and A/H3N2 viruses and the A(H7) HA genes were diagnosed using real-time PCR. Serological studies detected antibodies to A/H1N1pdm09 and A/H3N2. Our studies identified influenza isolates closely related to influenza A(H3N2) viruses recently circulating in humans, belonging to the J.2 or 3C.2a1b.2a.2a.3a.1 clade, suggesting potential introduction of viruses from humans into swine. The spread of influenza between humans and swine poses a significant risk of future pandemics. Therefore, monitoring swine influenza is essential to understand viral evolution and protect human health.

## 1. Introduction

Swine (Sus scrofa) are natural hosts of influenza viruses, which can cause extremely dangerous respiratory disease. Swine are susceptible to infection by all types of influenza viruses (IV)s A, B, C, and D [1]. Of all the IV types, IAVs are the most important pathogens posing a significant risk of zoonotic infection, host shifts, and the emergence of pandemic virus variants [2]. Although IAVs can infect a wide range of animals, including humans, wild and domestic birds, swine, horses, seals, whales, dogs, mink, and other mammals [3,4,5], the natural reservoirs of IAVs are wild birds (ducks, geese, swans, gulls, terns, and surfbirds) [6]. The main causative influenza viruses in swine are the influenza A virus (IAV) subtypes H1N1, H1N2, and H3N2 (family Orthomyxoviridae), which are antigenically related to humans [7]. This relationship is due to receptor specificity; tissues in the respiratory tract of pigs express both -2,6-sialic acid (-2,6-SA, the primary receptor for human IAV) and -2,3-SA (the primary receptor for avian IAV) [8,9]. Consequently, pigs can be co-infected with different viral lineages, creating an environment for genetic reassortment and the subsequent emergence of new strains with zoonotic potential.

A general trend in the ecology of IAVs is the periodic transmission of seasonal human influenza viruses into swine. The introduction of seasonal H1N1 and H3N2 IAVs from humans to swine at different times and in different geographic areas has led to the establishment of new IAV lineages and contributed to their genetic and antigenic diversity in swine hosts [10,11].

The first reports of swine influenza date back to the early 20th century, when J.S. Cohen described a new, highly contagious respiratory disease in swine in Iowa, which he called “flu” because of its clinical similarity to the Spanish flu pandemic in humans. He pointed out the difference between this disease and swine cholera and other known swine diseases and noted the coincidence of the swine disease with the influenza pandemic [12].

Confirmation of influenza circulation in swine in the United States was obtained in 1931, when Richard Shope isolated the first swine influenza virus [12]. Since then, IAVs (classical H1N1 and H3N2, their reassortants: H1N2, H3N1, H1N7, and avian: H9N2, H4N6) have been isolated from swine in various countries worldwide [6,13,14,15]. The A/H1N1pdm09 virus, which emerged in 2009 through host adaptation and reverse zoonosis, subsequently gave rise to various reassortants of swine influenza A viruses, including classical swine influenza A viruses, European avian influenza A viruses, and H3N2 viruses detected in swine worldwide [16,17,18,19,20].

The H3N2 virus was first identified in swine in 1970 following the 1968 influenza pandemic in humans. Its emergence is associated with interspecies transmission from humans to swine [21,22,23]. Then, H3N2 IAVs from swine were isolated in Taiwan (Asia) one year after the Hong Kong pandemic in 1970 [24]. Outbreaks of H3N2 IAV infection in swine have also been reported in Hong Kong, Japan, Korea, India, and Thailand [25,26,27,28,29,30,31,32,33,34]. Throughout the 1970s, 1980s, and 1990s, viruses similar to human strains (Victoria/75, Sydney/97, New York/99, and Moscow/99) were continually isolated from swine [35].

Although H3N2 IAVs are thought to be much less common among swine than A/H1N1, the situation has recently changed, and swine infections with this influenza virus have been recorded in many regions of the world, particularly in the United States and Asia. Since the mid-1980s, there have been five separate transmissions of seasonal H3N2 viruses in Brazil (three times in the late 1990s, once around 2011, and once around 2015). These human-origin viruses, established in swine, continue to evolve and become antigenically distinct from the parent viruses, resulting in a huge genetic diversity of swine IAVs (swIAVs). Phylogenetic analysis has identified three H3 genetic clades. The N2 segment of human seasonal H1N2 and H3N2 IAVs has been detected in swine six times [36].

Around the same time, double-reassortment viruses containing genes from the human (hemagglutinin (HA) and neuraminidase (NA)) and avian (PB2, PB1, PA, NP, M, and NS) lineages, as well as triple-reassortment viruses containing genes from the human (HA and NA), classical swine (NP), and avian (PB2, PB1, PA, M, and NS) lineages, began to appear in swine [21].

Swine influenza A(H3N2) viruses in China from 1970 to 2006 evolved from a 1968 human-like virus into various lineages, including triple reassortant strains. Frequent transmission from humans to swine, particularly in southern China, led to persistent infection and genetic reassortment, with viruses harboring human-like HA/NA genes alongside internal genes from avian or classical swine lineages [35]. Similar infections have been observed in Korea [33,37], Sri Lanka [38], Laos [39], and Cambodia [40].

In 1993, the virus was isolated in Japan [41], several outbreaks of respiratory disease were recorded in swine farms in America in 1998–1999 [42], reassortant variants of A/H3N2 were recorded in China from 1999 to 2002 [10], as well as cases of virus transmission between humans and swine [43,44,45,46,47]. Of particular concern recently are variant strains of the H3N2 virus (H3N2v), which periodically cause outbreaks among people in contact with swine at agricultural exhibitions. Although their transmission from person to person remains limited, the virus continues to accumulate mutations that facilitate its adaptation to the human host [48].

In the United States, repeated introductions of human H3N2 IAVs into the swine population since 1998 have resulted in the formation of distinct phylogenetic clusters I–IV [49,50,51,52,53], which have persisted and evolved into distinct phylogenetic clades [54].

Several outbreaks of respiratory disease with H3N2 IAVs in swine herds in North Carolina, Texas, Minnesota, and Iowa in 1998 identified two distinct pathways for the emergence of H3N2 IAVs in swine, mediated by genetic reassortment with both human and classical swine IAVs [50,55,56,57,58,59].

Most thoroughly studied swine viruses are characterized by the presence of an internal genetic element, TRIG (triple-reassortant internal gene), regardless of subtype. Therefore, reassortant H1 TRIG viruses remain endemic and circulate in swine-producing areas of the USA and Canada alongside other variants, such as H3N2 [21,42,49,50,53], H1N2 [59,60], and recombinant IV rH1N1 [50,61,62,63,64]. Reassortant swine H3-cluster 2010.1 viruses of the H1 lineage containing TRIG since 2012 differ significantly from earlier H3N2 strains and human seasonal H3N2 viruses, demonstrating continuous evolutionary progress in this lineage [65]. The zoonotic risks of swine influenza viruses to human health were demonstrated by the 2009 pandemic. This event was triggered by the emergence of a novel pandemic H1N1 strain containing swine-origin gene segments that had initially reassorted from human lineages, coinciding with a major swine influenza epizootic in Mexico City [66,67,68].

## 2. Materials and Methods

### 2.1. Sample Collection

**Figure 1:**
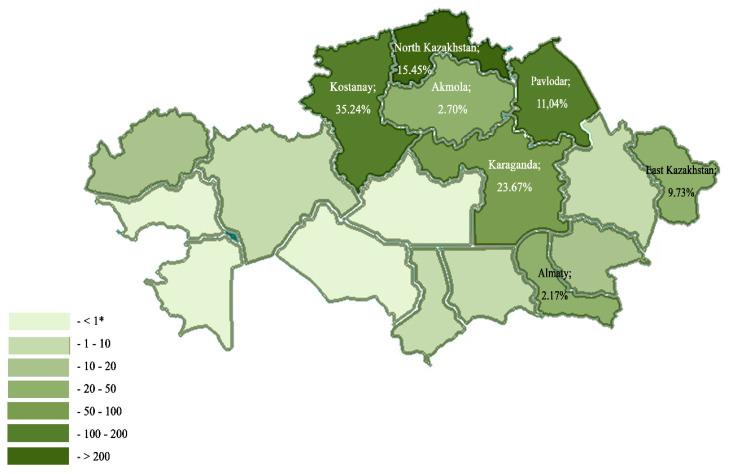
Geographic distribution of swine farms and percentage of samples collected. * Number of swine in various regions of Kazakhstan (thousands).

Biological samples, including nasopharyngeal swabs (*n* = 2031) and blood serum (*n* = 415), were obtained from swine between 2022 and 2025. The sampling was conducted at large-scale commercial swine production farms (ranging in capacity from 1000 to 30,000 animals) across various geographic regions of Kazakhstan. Figure 1 shows the geographic distribution of swine farms and the percentage of samples collected in each region.

The collection of biological samples was carried out in collaboration with licensed veterinarians in strict accordance with international veterinary and biosafety guidelines [69,70,71] and in accordance with the principles established by the Research and Ethics Committee of the International Association for the Study of Pain, and the study was approved by the local ethics committee (Protocol 20, 4 May 2026). Farm owners also provided informed consent for sample collection. During sample collection, every effort was made to minimize any pain or discomfort experienced by the animals.

Due to strict on-farm biosecurity protocols and veterinary regulations, a convenience sampling strategy based on site availability was employed. Animals without visible clinical signs of infectious or systemic diseases were selected from accessible herds. Furthermore, on-farm veterinarians limited sample collection to the finishing age group only, namely animals aged 1.5 to 6 months. To account for potential seasonal fluctuations in pathogen circulation, sampling was conducted twice a year (usually in spring and fall). Over the four-year monitoring period, a total of 2446 biological samples were successfully collected. The distribution of collected samples (nasopharyngeal swabs/blood serum) by year was as follows:

2022: 514 samples (465 swabs/49 serum);

2023: 350 samples (298 swabs/52 serum);

2024: 719 samples (553 swabs/166 serum);

2025: 863 samples (715 swabs/148 serum).

Nasopharyngeal swabs were collected using a sterile cotton swab on a sterile plastic handle into sterile tubes containing 1 mL of minimal essential medium (Gibco, Invitrogen, Grand Island, NY, USA). The resulting clinical specimens were stored at 4 °C and transported refrigerated to the laboratory for virological analysis within 48–72 h. Before analysis, specimens were vortexed for 15 s, centrifuged at 1000× *g* for 10 min at 4 °C, and the remaining specimens were stored in an ultra-low temperature freezer (≤80 °C).

Blood was collected in the morning before feeding from the tail vein using a vacuum collection system. Serum was separated by centrifugation at 400 g for 15 min. All serum samples were then transferred to new tubes and stored at −20 °C until analysis.

### 2.2. Real-Time Polymerase Chain Reaction

Viral RNA was isolated using the QIAamp Viral RNA Mini Kit (Qiagen, Hilden, Germany) according to the manufacturer’s instructions. cDNA synthesis of viral (v)RNAs was performed using SuperScript II reverse transcriptase (Gibco, Invitrogen, Grand Island, NY, USA) and random hexamers. Detection and typing/subtyping of influenza viruses were performed by real-time PCR using the SuperScript III Platinum^®^ One-Step qRT-PCR System (Gibco, Invitrogen, Grand Island, NY, USA) on a Rotor-Gene Q6 plex instrument (QIAGEN, Hilden, Germany).

Detection of Influenza A and B viruses, as well as subtyping of IAV (H1N1pdm09, H3N2, H5, H7, and H9), was performed using rtRT-PCR. The design of oligonucleotide primers and dual-labeled probes was adapted from the established protocols of the WHO and the US Centers for Disease Control and Prevention (CDC) [72,73]. The complete list of oligonucleotide sequences is summarized in Table 1.

Inactivated influenza virus strains were utilized as positive controls for both the extraction and amplification steps. Nuclease-free water was employed as a negative template control to rule out reagent contamination. To monitor sample collection quality, RNA extraction efficiency, and PCR inhibition, the human RNase P (RP) gene was used as an internal positive control.

The optimized one-step rtRT-PCR thermocycling profile consisted of an initial reverse transcription step at 50 °C for 15 min, followed by polymerase activation and initial denaturation at 95 °C for 5 min. This was followed by 45 cycles of denaturation at 95 °C for 10 s, and combined annealing/extension at 55 °C for 20 s. Fluorescence data were acquired during the annealing step via the Green, Yellow, and Orange optical channels.

### 2.3. Serological Screening of Blood Serum

Specific antibodies to HA IAV in serum were determined by the hemagglutination inhibition (HI) test using commercial diagnostic kits: A/Michigan/45/2015 (H1N1)pdm, A/Singapore/INFIMH-16-0019/2016 (H3N2), B/Phuket/3073/13 and B/Colorado/06/2017, and for influenza A/H5N1 and H7N9 viruses (Federal State Budgetary Institution “Research Institute of Influenza” of the Ministry of Health and Social Development of the Russian Federation, St. Petersburg). For HI antibody testing, chicken erythrocytes were used. Prior to testing, serum samples were treated with the respective erythrocytes to remove nonspecific agglutinins. To eliminate nonspecific agglutination inhibitors, receptor-destroying enzyme II (RDE II, Denka Co., Ltd., Tokyo, Japan) was used at a working dilution of 1:50. Serum was mixed with three volumes of RDE and incubated at 37 °C for 18–20 h. To achieve a final serum dilution of 1:10, six volumes of phosphate-buffered saline (PBS, pH 7.2) were added, followed by heat inactivation at 56 °C for 30 min.

Competitive ELISA was performed using test systems containing nucleoprotein-specific monoclonal antibodies to IAV (IDEXX Influenza A, Westbrook, ME, USA) according to the manufacturer’s instructions. The reaction involves competition for binding between nucleoprotein-specific monoclonal antibodies and, if present, serum antibodies (if present) and the highly conserved nucleoprotein of the virus, thereby determining the presence of influenza A infection. After the addition of the enzymatic substrate, the color intensity is inversely proportional to the concentration of antibodies to IAVs. To determine the presence or absence of antibodies to IAVs, the mean values of the negative (NCx) and positive (PCx) controls were calculated (the criteria for compliance were NCx ≥ 0.600 and PCx < 0.50), and the sample-to-negative ratio (S/N) was determined for each sample. A value of S/N < 0.60 was considered positive for the presence of antibodies to IAV NP in serum, and S/N ≥ 0.60 was considered negative.

### 2.4. Isolation of IAVs

Isolation of IAVs was performed by inoculating PCR-positive samples into 9- to 10-day-old chicken embryos and Madin-Darby canine kidney (MDCK) cells as described previously [74].

### 2.5. Sequence and Phylogenetic Analysis

Amplification of the entire IV genome was performed by one-step reverse transcription-polymerase chain reaction using the Biomaster RT-PCR-RV (2×) RT-PCR reagent kit (Biolabmix, Novosibirsk, Russia) according to modified protocols for IAV [75,76] on Bio-Rad CFX96 Touch systems. The PCR products (fragments) obtained after amplification of the entire IV genome were verified by electrophoresis on a 2% agarose gel in TAE buffer.

Libraries for whole-genome sequencing were prepared according to the manufacturer’s instructions using the Illumina DNA Prep kit (Illumina, San Diego, CA, USA). Samples were indexed using the Illumina IDT^®^ Illumina^®^ DNA/RNA UD Indexes Set A/B/C/D (Illumina, San Diego, CA, USA). AMPure XP magnetic beads (Beckman, Indianapolis, IN, USA) were used to purify whole-genome amplification products for whole-genome sequencing. The concentration of double-stranded DNA in PCR products was measured using the Qubit HS dsDNA kit (Life Technologies, Carlsbad, CA, USA) on a Qubit 4 fluorometer (Thermo Fisher Scientific, Waltham, MA, USA). Purified cDNA samples were diluted to a working concentration of 0.2 ng/µL, and libraries were then prepared using Illumina Nextera XT reagent kits (Illumina, San Diego, CA, USA). Sequencing was performed on an Illumina NextSeq 2000 sequencer (Illumina, San Diego, CA, USA) using the Illumina NextSeq P2 100-cycle kit (Illumina, San Diego, CA, USA).

For phylogenetic analysis, multiple alignments of HA and NA gene sequences were performed using the ClustalW algorithm in MEGA 12 software (version 12.1.2). For comparative analysis, closely related, reference, and vaccine strains of A(H3N2) were obtained from the international GISAID (13 February 2026) and GenBank (16 February 2026) databases (Supplementary Materials). Phylogenetic analysis was performed using MEGA 12 software (version 12.1.2) (The Pennsylvania State University, University Park, PA, USA) [77,78]. The reliability of the tree topology was evaluated using the adaptive bootstrap method for Maximum Likelihood implemented in MEGA 12, with 1000 replicates [79]. Phylogenetic trees were constructed using the maximum likelihood (ML) method. To estimate evolutionary distances for the HA gene, the Hasegawa–Kishino–Yano model (1985) [80] was used; for NA, the Tamura 3-parameter model (1992) [81] was used. Accounting for heterogeneity in evolutionary rates across sites was performed using a 5-category discrete gamma distribution (k = 5). The estimated shape parameter for HA was (α = 0.30), which indicates a significant variation in substitution rates in the analyzed sequence, and for NA, the shape parameter was (α = 0.7), which may indicate a more uniform distribution.

Analysis of differences in HA and NA gene sequences (amino acid substitutions, insertions/deletions, nucleotide divergences, and potential N- and O-glycosylation sites) and clades classification were performed using the Nextclade v3.18.1 web interface (18 February 2026) [82].

### 2.6. Statistical Analysis

Statistical analysis was performed using GraphPad Prism version 9.1 software. Categorical variables were analyzed using Fisher’s exact test, a nonparametric method for calculating exact probabilities in small samples (*p*-values). The level of statistical significance was set at *p* < 0.05. Confidence intervals (CI) for proportions were calculated using the Clopper-Pearson exact method based on the binomial distribution for small sample sizes. Calculations were performed in Microsoft Excel 2016. The significance level was set to α = 0.05 (95% CI).

## 3. Results

### 3.1. Screening of Samples by rtRT-PCR

A total of 2031 nasopharyngeal swabs collected from 2022 to 2025 were subjected to real-time PCR; the results are presented in Table 2. A positive result for IAVs RNA was detected in 98 samples (3.92%). Subtyping of IAVs-positive samples revealed IAVs H1N1 RNA in 32 samples (1.58%), H3N2 in 22 cases (1.08%), and IAV with H7 HA in 9 samples (0.44%). The virus subtype could not be identified in 35 PCR-positive samples (1.72%). The results of the significance test for H1N1, H3N2, and H7 IAVs were statistically insignificant, whereas the number of analyzed samples and the number of PCR-positive samples for Influenza A and undetermined Influenza A were statistically significant. Taken together, the real-time PCR results for nasopharyngeal swabs indicate a predominance of A/H1N1 strains among IAVs circulating on swine farms in Kazakhstan during the study period.

### 3.2. Serological Analysis

**Figure 2:**
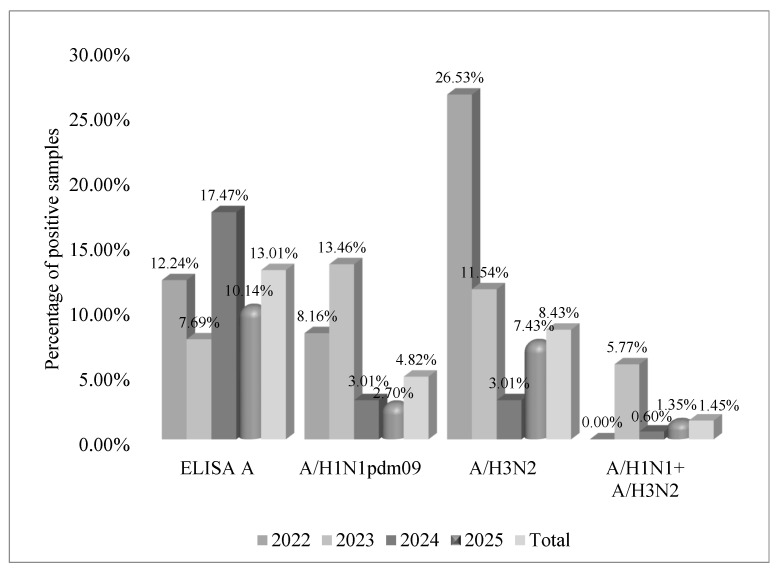
Results of serological screening of blood serum for antibodies using HI and ELISA.

The results of the serological analysis of 415 blood samples using HI and ELISA are presented in Figure 2.

As shown in Figure 2, of the 414 blood samples tested by ELISA, antibodies to IAVs were detected in 13.01% (*n* = 54). The HI test results revealed the presence of antihemagglutinins to A/H1N1pdm in 4.82% of cases (20 samples) of the total number of samples; antihemagglutinins to A/H3N2 in 8.43% (35 samples); and antihemagglutinins to both IAV (H1N1pdm and H3N2) in 1.45% (six samples). Antibodies to IAVs H5, H7, and type B IV were not detected. Antibody titers ranged from 1:40 to 1:160.

Thus, ELISA allowed us to detect antibodies to IAVs, while the HI method detected antibodies to the H1 (including the pandemic strain A(H1N1)pdm09) and H3 subtypes. However, the methods used did not allow for neuraminidase typing; therefore, the possibility of circulating reassortant strains cannot be ruled out. In addition, because H3N2 viruses undergo rapid antigen drift, the use of the 2016 reference antigen may significantly understate seroprevalence due to antigen mismatch.

### 3.3. Isolation of IAVs from Swine

Inoculation of 98 PCR-positive samples into MDCK cell culture and 9- to 10-day-old chicken embryos, followed by culture, identified two hemagglutinating agents with hemagglutination titers ranging from 1:2 to 1:32. Samples were collected in the Karaganda region.

IAV was identified using real-time RT-PCR and HI with diagnostic immune sera specific for reference IAV strains. Isolates from the Karaganda region (A/swine/Karaganda/36/2024 and A/swine/Karaganda/45/2024) belonged to the A/H3N2 subtype.

### 3.4. Sequencing

**Figure 3:**
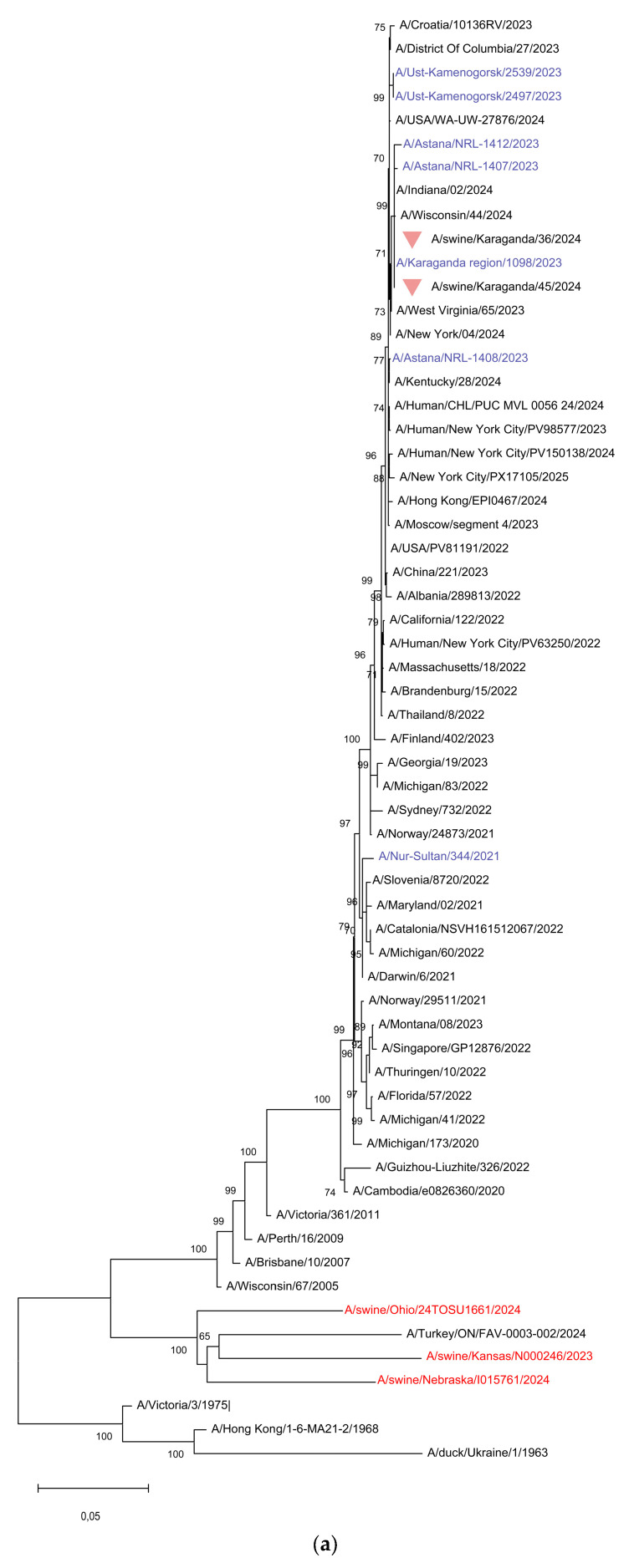

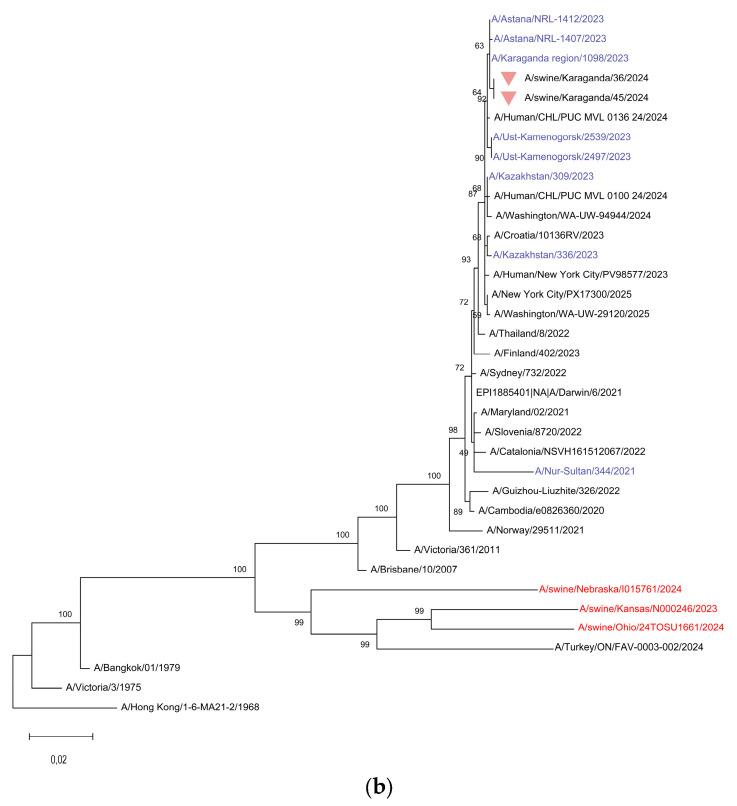
Molecular phylogenetic analysis of HA and NA is shown. Subclade classification was by maximum likelihood: (**a**) the tree for HA is inferred with the highest log-likelihood (–5087.16) using the Hasegawa-Kishino-Yano (1985) [80] model; (**b**) the tree for NA is inferred with the highest log-likelihood (–3668.78) using the Tamura (1992) model [81]. Numbers at nodes represent bootstrap support values from 1000 replicates. The pink triangles represent the nucleotide sequences of hemagglutinin and neuraminidase genes in swine samples from the Karaganda region. Viruses obtained in humans of Kazakhstan are highlighted in blue; swine H3N2 viruses are highlighted in red.

Direct sequencing of eight IV-positive PCR samples yielded the complete genome of the A/swine/Karaganda/36/2024 virus and partial sequences of the A/swine/Karaganda/45/2024 IV genome (the HA, NP, NA, MP, and NS genes). Molecular phylogenetic analysis for classification of the H3 subclade using the IAV H3N2 maximum likelihood method is presented in Figure 3a.

The results of phylogenetic analysis showed that the HA and NA genes are more closely related to IAV circulating among people in Kazakhstan (A/Astana/NRL-1412/2023, A/Astana/NRL-1407/2023, A/Karaganda region/1098/2023, A/Ust-Kamenogorsk/2539/2023, A/Ust-Kamenogorsk/2497/2023, A/Astana/NRL-1408/2023), in the Russian Federation (A/Moscow/segment 4/2023), in the USA (A/Indiana/02/2024, A/West Virginia/65/2023, A/New York/04/2024, A/USA/WA-UW-27876/2024, A/Croatia/10136RV/2023, A/Kentucky/28/2024), in China (A/Hong Kong/EPI0467/2024, A/China/221/2023), as well as with IAV strains circulating in different countries of the world. The analysis showed a close relationship to the A/District of Columbia/27/2023 and A/Croatia/10136RV/2023 strains recommended for use in vaccines for the 2025 influenza season, as well as to the reference strains A/Thailand/8/2022 and A/Massachusetts/18/2022 recommended by the World Health Organization for vaccines in 2024–2025 in the Southern Hemisphere (Figure 3).

The A/swine/Karaganda/36/2024 and A/swine/Karaganda/45/2024 viruses belonged to clade 2a.3a.1 and, relative to the A/Darwin/6/2021 vaccine virus, showed differences in 12 amino acid sequences of the HA gene (HA1: E50K, G53N, N96S, N122D, I140K, I192F, I223V, K276E, N49S, HA2: L80I, and in the region of the SigPep:T3A signal peptides). Five amino acid substitutions (E83G, R150H, D221N, T247A, R400K) were also found in the NA gene sequence of the virus relative to the vaccine strain A/Darwin/6/2021, of which two were unique (D221N and T247A).

In addition, mutations were found in other viral genes. The following substitutions were identified: one substitution in the M1 protein in MP (V219I), five substitutions in M2 (P25L, S31N, L54F, V68I, N82S), six substitutions in NP (A131S, I136L, V197I, R236K, G384R, T472A), six substitutions in the NS1 sequence (E26N, L33I, V60A, V82A, M124I, I171V), two in PB1-F2 (Q5L, R21E), four in PB2 (S107N, I147T, S490N, R500K), four in PA (N142K, I311M, Y321C, K605R) and three in the PA-X protein gene (N142K, E209G, S236L). The S31N mutation identified in the M2 gene may indicate virus resistance to the antiviral drug rimantadine. However, most currently circulating influenza virus strains have this mutation in the M2 protein, rendering these drugs ineffective and not recommended for the treatment of influenza A. Current CDC and WHO guidelines recommend the use of neuraminidase inhibitors (oseltamivir, etc.) and ceiling-dependent endonexulin inhibitors (baloxvir marbokil) for the treatment of influenza. Resistance to these drugs has not been identified.

The sequences of A/swine/Karaganda/36/2024 and A/swine/Karaganda/45/2024 were registered in the GISAID database under the numbers EPI_ISL_19186454 and EPI_ISL_19186455, respectively, and in NCBI (numbers PX589477 and PX599200).

## 4. Discussion

Swine farms in Kazakhstan are distributed unevenly geographically (Figure 1). The North Kazakhstan (266.9 thousand heads), Kostanay (136.9 thousand heads), and Pavlodar (110.7 thousand heads) regions have the largest swine populations [83]. This is due to climatic features and regional differences in meat consumption. The main differences are the high popularity of pork in the northern/eastern regions and among the Slavic population, while in the southern regions, consumption is minimal due to religious and cultural traditions. Therefore, in the southern and western regions (Aktobe, Mangystau, Atyrau, Kyzylorda, and others), swine farming is underdeveloped.

Samples were collected taking into account the geographic distribution of the swine population: the majority of our samples were collected in northern Kazakhstan (North Kazakhstan, Kostanay, and Pavlodar regions), where the swine farming industry is most developed (61.73%), while smaller numbers of samples were obtained in central Kazakhstan (23.67%), eastern Kazakhstan (9.73%), and southern Kazakhstan (2.17%). Unfortunately, no samples were obtained in western Kazakhstan.

**Figure 4:**
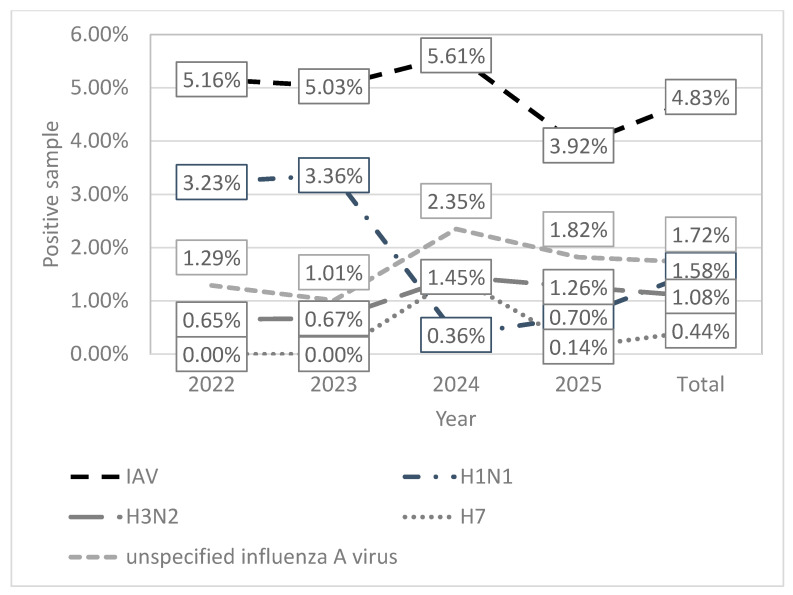
Percentage of swine positive for the influenza virus in Kazakhstan for 2022–2025.

The dynamics of IAV circulation among swine in Kazakhstan during the study period are shown in Figure 4, which indicates that IAV detection ranged from 3.92% to 5.61%, with the majority being the A/H1N1 subtype (1.58%). However, in 2024 and 2025, the positive sample rate decreased to 0.36% and 0.70%, respectively. Nevertheless, this subtype was detected most frequently, accounting for 1.91% of the total number of positive samples. For unidentified IAV, the percentage of positive samples was slightly higher (1.72%).

**Figure 5:**
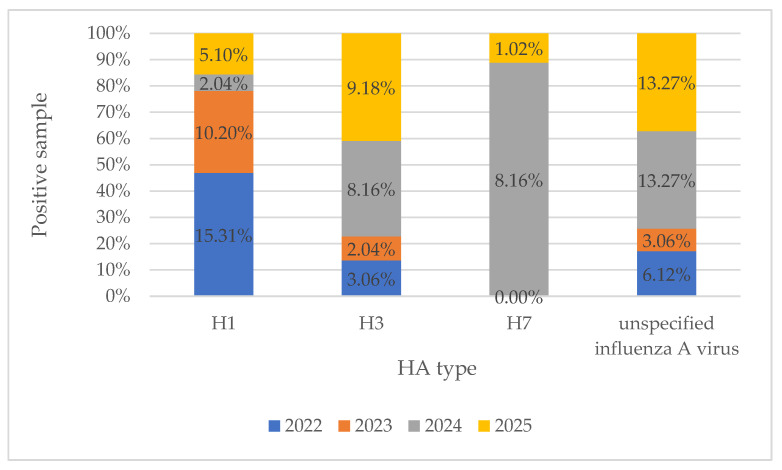
Percentage of positive samples depending on HA type.

The percentage of samples containing A/H1 genetic material decreased from 15.31% of the total number of positive samples in 2022 to 10.20% in 2023, then to 2.04% in 2024, and then increased again to 5.10% in 2025 (Figure 5). For IV A/H3, these indicators decreased from 3.06% in 2023 to 2.04% in 2022, then increased to 8.16% and 9.18% in 2024 and 2025, respectively. Positive samples to IV A/H7 were detected only in 2024 (8.16%) and 2025 (1.02%).

Introductions of IAV from humans to swine occur regularly, making a significant contribution to the evolution of swine influenza. Events of reverse zoonoses of A(H3N2) viruses from humans to swine, associated with unexpected clinical consequences on farms raising young animals, were identified in Chile [84]. In the United States, the epidemiology of IAVs in swine changed dramatically after 1998, when triple reassortants of the H3N2 virus appeared in the swine population, containing gene segments from cH1N1 (NP, M, NS), human seasonal IAV subtype H3N2 (PB1, HA, NA), and avian IAV (PB2, PA) [21,55]. In 2010–2020, cases of transmission of the H3 virus from humans to swine were recorded, which, as a result of adaptation, became established in swine with stable transmission. The nucleotide sequence identity of human H3 IAVs (H3.2010.2) detected in swine with the 2016–2017 human seasonal H3 was 99.9% [85].

In Kazakhstan, data on the spread of A/H1N1 IAV among swine have been previously published. In 1984, three strains of classical swine A/H1N1 were isolated from swine in eastern Kazakhstan for the first time [1,86]. Subsequently, swine IAVs H1N1 were systematically identified in various parts of the country [87]. Molecular and serological analyses of samples collected from farms across various regions of Kazakhstan in 2018–2021 revealed the cocirculation of A/H1N1 and A/H3N2 viruses among animals [86]. In 2020, the complete genomic sequence of the IAV A/swine/Karaganda/04/2020 (H1N1) was obtained from a clinical sample from a swine on a livestock farm in Karaganda (Central Kazakhstan). The isolate belonged to clade 1A.3.2.2 of lineage 1A, which includes the 2009 pandemic H1N1 strains [88].

Phylogenetic analysis of the isolates A/swine/Karaganda/36/2024 and A/swine/Karaganda/45/2024 revealed that they belong to clade 2a.3a.1. Both IAVs retained a full set of internal genes originating from the human virus. Compared with the vaccine reference strain A/Darwin/6/2021, the sequences of all eight genomic segments of the Karaganda isolates exhibited specific mutations characteristic of the recently circulating human seasonal A(H3N2) viruses [89,90]. Based on phylogenetic analysis, it can be inferred that the influenza A (H3N2) virus may have been transmitted from humans to swine. However, without concomitant clinical isolates from the local human population or agricultural workers, it cannot be excluded that transmission from swine to humans or co-circulation from a common reservoir may occur.

Despite a comprehensive molecular and serological analysis of the circulation of influenza A/H1N1 and A/H3N2 viruses in swine in Kazakhstan, our study has several limitations. Because sample collection was conducted based on the geographic distribution of the swine population, unfortunately, it was not possible to obtain samples in Western Kazakhstan, despite the presence of swine farms in the region. Since livestock and epidemiological data can vary greatly by region, extrapolating estimates without representative data from Western Kazakhstan (Atyrau, Mangystau, West Kazakhstan, and Aktobe regions) may lead to statistical errors. Furthermore, infection statistics may underestimate the true scale of the disease due to its latent nature. Also, repeat sample collection was not conducted at all farms. Effective subtyping was not possible for all samples. The virus subtype was not identified in 35 PCR-positive samples (1.72%). This may have been due to several factors. First, the initial clinical samples had a low viral load (high Ct values). Second, storage and transportation conditions of the samples may have reduced virus viability (samples were stored at 4 °C and transported for 48–72 h before freezing, which may have reduced infectivity). Third, some PCR-positive samples may have contained nonviable or defective viral particles. Fourth, outdated probe sets may have been used. Future studies plan to cover a broader range of swine farms in Kazakhstan and use updated probe sets for subtyping.

In addition to the above-mentioned reasons, the inability to isolate viruses from clinical samples may be due to the low susceptibility of the cell lines and chicken embryos used to modern influenza viruses. This limited the amount of genetic information obtained. Since the phylogenetic analysis was based on only one complete and one partial genome, these two isolates cannot be considered representative of the entire genetic diversity of influenza A viruses circulating in the swine population across Kazakhstan.

To overcome these limitations, in future studies, we plan to use high-throughput next-generation sequencing (NGS) directly from clinical samples, bypassing the need for virus isolation. Future studies plan to cover a wider range of swine farms in Kazakhstan and use updated probe sets for subtyping.

Also, phylogenetic analysis based on only one complete and one partial genome means that these two isolates cannot be considered representative of the entire genetic diversity of influenza A virus circulating in the swine population throughout Kazakhstan. To overcome these limitations, we plan to use high-throughput next-generation sequencing (NGS) directly from clinical samples in future studies, bypassing the need for virus isolation.

A negative HI result for H5 and H7, coupled with a positive PCR result, may be due to blood samples being collected early in the infection process (7–14 days), before antibody production. Furthermore, the high sensitivity of PCR allows the detection of non-replicating viral RNA or defective viral particles in nasopharyngeal swab samples, whereas the high specificity of HI does not detect mutated viral variants. Given that many viruses undergo rapid changes in antigenic structure, the use of more modern reference antigens is planned for the future.

Despite the negative HAI result for H5 and H7, the detection of H7 RNA in swine was noteworthy. However, the lack of sequencing data in this study precludes definitive confirmation of the virus’s phylogenetic origin and pathogenicity. Given the endemic nature of avian influenza in the study region, we hypothesize that H7 detection may result from interspecies transmission between wild and domestic birds. However, these results should be considered preliminary. Further targeted studies, including mandatory whole-genome sequencing, are urgently needed to confirm H7 circulation in swine populations and assess potential pandemic risks.

Thus, various IAV variants (H1N1, H3N2, H7, and an unknown subtype) circulate among swine in Kazakhstan. Despite the absence of confirmed cases of reassortant viruses in the swine population, the likelihood of their emergence in the current context of IAV spread is increasing.

## 5. Conclusions

A RT-PCR study of swine biological material collected from livestock farms in various regions of Kazakhstan in 2022–2025 revealed the continued circulation of IAVs. Most influenza-positive samples belonged to the A/H1N1 subtype (0.36% in 2024, 3.36% in 2023), but there was a trend toward increasing infection of swine with H3N2 viruses, reaching 1.45% in 2024. The number of infected swine identified ranged from 3.92% to 5.61%. IAV circulation among the swine population was confirmed by the isolation of two A/H3N2 strains. Health officials confirm that human-like IAVs adapted to swine continue to pose a zoonotic infection threat. A clear example is the H3.2010.1 lineage, which, after six years of circulation in swine, has become the main cause of zoonotic influenza infection in humans [85,91,92]. The obtained data on the infection of swine with H7 IAVs in 0.44% of cases complement the information available worldwide and in Kazakhstan and confirm the complex interaction between the ecology of IAV, host specificity, and the dynamics of virus transmission between humans and swine, their persistence in swine populations, facilitating the emergence of genetic reassortants and the subsequent transmission of reassortant viruses to humans. These processes of IAV spread between human and swine populations are significant risk factors for future pandemics. In this regard, continuous monitoring of swine influenza infection with genomic surveillance, especially in the reproductive and fattening sectors, which are most susceptible to a high risk of persistent infection, is necessary for an in-depth study of IAV evolution and is of great importance both for the protection of animal health and for the prevention of future pandemics in humans [84,92]. 

## Figures and Tables

**Table 1:** Oligonucleotide sequences of primers and probes used for the rtRT-PCR detection and subtyping of Influenza viruses.

Type/Subtype	Gene/Target	Name	Primer/Probe
*Influenza* *A virus*	*M*	FLUAM-7-F	CTTCTAACCGAGGTCGAAACGTA
		FLUAM-161-R	GGTGACAGGATTGGTCTTGTCTTTA
		FLUAM-49-P6	TCAGGCCCCCTCAAAGCCGAG
*Influenza* *B virus*	*HA*	FLUBHA-940-F	AAATACGGTGGATTAAACAAAAGCAA
		FLUBHA-1109-R	CCAGCAATAGCTCCGAAGAAA
		FLUBHA-994-P4	CACCCATATTGGGCAATTTCCTATGGC
*Influenza A(H1N1)pdm09*	*HA*	H1pdm-169-F	AAACTATGCAAACTAAGAGGGGT
		H1pdm-297-R	TGTTTCCACAATGTAGGACCA
		H1pdm-244-P	CCAGAGTGTGAATCACTCTCCACA
*A(H3)*	*HA*	H3-266-F	ACCCTCAGTGTGATGGCTTTCAAA
		H3-373-R	TAAGGGAGGCATAATCCGGCACAT
		H3-315-P	ACGAAGCAAAGCCTACAGCAACTGTT
*A(H5)*	*HA*	H5-1201F	CARGGGAGTGGDTAYGCBGCAGA
		H5-1387R	ARAAGTTCAGCRTTRTARGTCCA
		H5-1285P	AARATGAACASTCARTTYGAGG
*A(H7)*	*HA*	NIID-H7 TMPrimer-F1	TGTGATGAYGAYTGYATGGCCAG
		NIID-H7 TMPrimer-R1	ACATGATGCCCCGAAGCTAAAC
		NIID-H7 Probe1	ATCTGTATTCTATTTTGCATTGCYTC
*A(H9)*	*HA*	H9-1538-F	GGGTCAAGCTGGAATCTGA
		H9-1651-R	TGGACATGGCCCAGAACAAGAA
		H9-1567p2	GTCGCCTCATCTCTTGTGVTTGCAA
Internal control	*RP (Human PH)*	RP-F	AGATTTGGACCTGCGAGCG
		RP-R	GAGCGGCTGTCTCCACAAGT
		RP-P	TTCTGACCT/TAO/GAAGGCTCTGCGCG

**Table 2:** Results of rtRT-PCR of nasopharyngeal swabs collected from pigs.

Sample Collection Year	Number of Analyzed Samples	Number/Percentage of PCR-Positive Samples (Confidence Intervals)				
		Influenza A				Subtype
			A/H1N1	A/H3N2	H7	Undetermined
Total	2031	98/3.92 (3.93; 5.85)	32/1.58 (1.08; 2.22)	22/1.08 (0.68; 1.64)	9/0.44 (0.20; 0.84)	35/1.72 (1.20; 2.39)
2022	465	24/5,16 (3.33; 7.58)	15/3.23 (1.82; 5.26)	3/0.65 (0.13; 1.87)	0	6/1.29 (0.47; 2.79)
East Kazakhstan region	50	4/8.00 (2.22; 19.23)	1/2.00 (0.05; 10.65)	1/2.00 (0.05; 10.65)	0	2/4.00 (0.49; 13.71)
Karaganda region	145	8/5.52 (2.41; 10.58)	7/4.83 (1.96; 9.69)	0	0	1/0.69 (0.02; 3.78)
Kostanay region	140	7/5.00 (2.03; 10.03)	5/3.57 (1.17; 8,14)	1/0.71 (0.02; 3,92)	0	1/0.71 (0.02; 3,92)
North Kazakhstan region	130	5/3.85 (1.26; 8.75)	2/1.54 (0.19; 5.45)	1/0.77 (0.02; 4.21)	0	2/1.54 (0.19; 5.45)
2023	298	15/5.03 (2.84; 8.17)	10/3.36 (1.62; 6.08)	2/0.67 (0.08; 2.40)	0	3/1.01 (0.21; 2.91)
Karaganda region	50	2/4.00 (2.22; 19.23)	1/2.00 (0.05; 10.65)	0	0	1/2.00 (0.05; 10.65)
Kostanay region	100	5/5.00 (1.64; 11.28)	3/3.00 (0.62; 8.52)	0	0	2/2.00 (0.24; 7.04)
North Kazakhstan region	50	1/2.00 (0.05; 10.65)	1/2.00 (0.05; 10.65)	0	0	0
Pavlodar region	98	7/7.14 (2.92; 14.16)	5/5.10 (1.68; 11.51)	2/2.04 (0.25; 7.18)	0	0
2024	553	31/5.61 (3.84; 7.86)	2/0.36 (0.04; 1.30)	8/1.45 (0.63; 2.83)	8/1.45 (0.63; 2.83)	13/2.35 (1.26; 3.99)
Akmola Region	50	3/6.00 (1.25; 16.55)	1/2.00 (0.05; 10.65)	1/2.00 (0.05; 10.65)	0	1/2.00 (0.05; 10.65)
Almaty Region	53	8/15.09 (6.75; 27.59)	0	2/3.77 (0.46; 12.98)	4/7.55 (2.09; 18.21)	2/3.77 (0.46; 12.98)
Karaganda region	150	10/6.67 (3.24; 11.92)	0	2/1.33 (0.16; 4.73)	4/2.67 (0.73; 6.69)	4/2.67 (0.73; 6.69)
Kostanay region	150	7/4.67 (1.90; 9.38)	0	2/1.33 (0.16; 4.73)	0	5/3.33 (1.09; 7.61)
East Kazakhstan region	50	2/2.00 (0.49; 13.71)	0	1/2.00 (0.05; 10.65)	0	1/2.00 (0.05; 10.65)
North Kazakhstan region	100	1/1.00 (0.03; 5.45)	1/1.00 (0.03; 5.45)	0	0	0
2025	715	28/3.92 (2.62; 5.61)	5/0.70 (0.23; 1.62)	9/1.26 (0.58; 2.38)	1/0.14 (0.004; 0.78)	13/1.82 (0.97; 3.09)
East Kazakhstan region	103	4/3.88 (1.07; 9.65)	0	2/1.94 (0.24; 6.84)	0	2/1.94 (0.24; 6.84)
Karaganda region	182	7/3.85 (1.56; 7.76)	1/0.55 (0.01; 3.02)	3/1.65 (0.34; 4.74)	0	3/1.65 (0.34; 4.74)
Kostanay region	204	9/4.05 (2.04; 8.21)	1/0.49 (0.01; 2.70)	2/0.98 (0.12; 3.50)	1/0.49 (0.01; 2.70)	5/2.45 (0.80; 5.63)
North Kazakhstan region	54	2/3.70 (0.45; 12.75)	0	1/1.85 (0.05; 9.89)	0	1/1.85 (0.05; 9.89)
Pavlodar region	172	6/3.49 (1.29; 7.44)	3/1.74 (0.36; 5.01)	1/0.58 (0.01; 3.20)	0	2/1.16 (0.14; 4.14)
*p* value	0.02069 *	0.021644	0.162480	0.095312	0.328185	0.027207

* *p* ≤ 0.05 was considered statistically significant.

## Data Availability

The consensus sequences of the viruses analyzed in this study were submitted to the GISAID EpiFlu™ and NCBI databases under the accession numbers reported in the last paragraph of Section 3.

## Supplementary Materials

The following supporting information can be downloaded at: https://www.mdpi.com/article/10.3390/ani16111752/s1, Table S1: List of GISAID and GenBank access numbers used as reference data, as indicated in the manuscript.

## Abbreviations

The following abbreviations are used in this manuscript:

IAV	Influenza A virus
IV	Influenza virus
rtRT-PCR	Real-time polymerase chain reaction
ELISA	Enzyme-linked immunosorbent assay
HI	Hemagglutinating activity inhibition
