# Supplementary material for: Human-Origin Influenza A(H3N2) Viruses Revealed in Swine Farms During the Period 2022–2025 in Kazakhstan
Source: Animals (Basel). 2026 Jun 5;16(11):1752. doi: 10.3390/ani16111752 (PMC13255645; doi:10.3390/ani16111752)
Supplement: Supplementary file 1 [file animals-16-01752-s001.zip › animals-4336658-supplementary.pdf]

Table S1. List of GISAID and GenBank access numbers used as reference data, as indicated in the manuscript.

Name	GISAID/GenBank access numbers	Clade
A/Astana/NRL-1407/2023 (H3N2)	EPI_ISL_19458417	2a.3a.1
A/Karaganda region/1098/2023 (H3N2)	EPI_ISL_18872896	2a.3a.1
A/Wisconsin/44/2024 (H3N2)	EPI_ISL_19050734	2a.3a.1
A/West Virginia/65/2023	PP690055.1	2a.3a.1
A/New York/04/2024 (H3N2)	PP683573.1	2a.3a.1
A/USA/WA-UW-27876/2024 (H3N2)	PP815304.1	2a.3a.1
A/Croatia/10136RV/2023 (H3N2)	EPI_ISL_20414456	2a.3a.1
A/District Of Columbia/27/2023 (H3N2)	PP684854.1	2a.3a.1
A/Human/CHL/PUC MVL 0056 24/2024 (H3N2)	PX051620.1	2a.3a.1
A/Human/New York City/PV98577/2023 (H3N2)	PV062570.1	2a.3a.1
A/Human/New York City/PV150138/2024 (H3N2)	PQ068511.1	2a.3a.1
A/New York City/PX17105/2025 (H3N2)	PV885832.1	2a.3a.1
A/Hong Kong/EPI0467/2024 (H3N2)	PP535504.1	2a.3a.1
A/Moscow/segment 4/2023 (H3N2)	PQ632958.1	2a.3a.1
A/Astana/NRL-1408/2023 (H3N2)	EPI_ISL_19448033	2a.3a.1
A/Kentucky/28/2024 (H3N2)	PQ263873.1	2a.3a.1
A/USA/PV81191/2022 (H3N2)	OQ842187.1	2a.3a.1
A/China/221/2023 (H3N2)	PP478135.1	2a.3a.1
A/Albania/289813/2022 (H3N2)	EPI_ISL_17953387	2a.3a.1
A/Thailand/8/2022 (H3N2)	EPI_ISL_16014504	2a.3a.1
A/California/122/2022 (H3N2)	EPI_ISL_17832066	2a.3a.1
A/Human/New York City/PV63250/2022 (H3N2)	OR042365.1	2a.3a.1
A/Brandenburg/15/2022 (H3N2)	EPI_ISL_17953386	2a.3a.1
A/Massachusetts/18/2022 (H3N2)	EPI_ISL_16998756	2a.3a.1
A/swine/Karaganda/36/2024	EPI_ISL_19186454	2a.3a.1
A/swine/Karaganda/45/2024	EPI_ISL_19186453	2a.3a.1
A/Ust-Kamenogorsk/2539/2023	EPI_ISL_19186461	2a.3a.1
A/Ust-Kamenogorsk/2497/2023	EPI_ISL_19186465	2a.3a.1
A/Finland/402/2023 (H3N2)	EPI_ISL_18237981	2a.3a
A/Georgia/19/2023 (H3N2)	EPI_ISL_17832073	2a.3
A/Michigan/83/2022 (H3N2)	EPI_ISL_17832070	2a.3
A/Norway/24873/2021 (H3N2)	EPI_ISL_12240180	2a.3
A/Sydney/732/2022 (H3N2)	EPI_ISL_16003445	2a.3b
A/Nur-Sultan/344/2021 (H3N2)	EPI_ISL_12809003	2a.2
A/Darwin/6/2021 (H3N2)	EPI_ISL_3534319	2a
A/Slovenia/8720/2022 (H3N2)	EPI_ISL_12943148	2a.1
A/Maryland/02/2021 (H3N2)	EPI_ISL_11140790	2a.1a
A/Catalonia/NSVH161512067/2022 (H3N2)	EPI_ISL_16043979	2a.1b
A/Michigan/60/2022 (H3N2)	EPI_ISL_17832067	2a.1b
A/Norway/29511/2021 (H3N2)	EPI_ISL_11140795	2
A/Michigan/173/2020 (H3N2)	MW796047.1	2
A/Florida/57/2022 (H3N2)	EPI_ISL_16947030	2b
A/Michigan/41/2022 (H3N2)	EPI_ISL_16947039	2b
A/Thuringen/10/2022 (H3N2)	EPI_ISL_13704107	2b
A/Montana/08/2023 (H3N2)	EPI_ISL_17832071	2b
A/Singapore/GP12876/2022 (H3N2)	EPI_ISL_17374548	2b

A/Guizhou-Liuzhite/326/2022 (H3N2)	EPI_ISL_14863099	1a.1
A/Cambodia/e0826360/2020 (H3N2)	EPI_ISL_806547	1a
A/Victoria/361/2011 (H3N2)	KM821347.1	3C
A/Perth/16/2009 (H3N2)	GQ293081.1	unassigned
A/Brisbane/10/2007 (H3N2)	EU199250.1	unassigned
A/Wisconsin/67/2005 (H3N2)	EF473424.1	unassigned
A/Victoria/3/1975 (H3N2)	EF626609.1	unassigned
A/Hong Kong/1-6-MA21-2/1968(H3N2)	HM641168.1	unassigned
A/swine/Nebraska/I015761/2024(H3N2)	EPI_ISL_19268400	unassigned
A/Turkey/ON/FAV-0003-002/2024	EPI_ISL_19804513	unassigned
A/swine/Ohio/24TOSU1661/2024	EPI_ISL_19332136	unassigned
A/duck/Ukraine/1/1963(H3N8)	GU052321.1	unassigned
